# Identification of Six Prognostic Genes in EGFR–Mutant Lung Adenocarcinoma Using Structure Network Algorithms

**DOI:** 10.3389/fgene.2021.755245

**Published:** 2021-11-16

**Authors:** Haomin Zhang, Di Lu, Qinglun Li, Fengfeng Lu, Jundong Zhang, Zining Wang, Xuechun Lu, Jinliang Wang

**Affiliations:** ^1^ Department of Hematology, The Second Medical Center, Chinese PLA General Hospital, National Clinical Research Center for Geriatric Disease, Beijing, China; ^2^ Department of Oncology, The Fifth Medical Center of Chinese PLA General Hospital, Beijing, China; ^3^ Medical School of Chinese PLA, Beijing, China; ^4^ College of Science, University of Shanghai for Science and Technology, Beijing, China; ^5^ State Key Laboratory of Toxicology and Medical Countermeasures, Beijing Key Laboratory of Neuropsychopharmacology, Beijing Institute of Pharmacology and Toxicology, Beijing, China

**Keywords:** EGFR–mutant lung adenocarcinoma, prognosis, WGCNA, TCGA, GEO

## Abstract

This study aims to determine hub genes related to the incidence and prognosis of EGFR-mutant (MT) lung adenocarcinoma (LUAD) with weighted gene coexpression network analysis (WGCNA). From The Cancer Genome Atlas (TCGA) and Gene Expression Omnibus (GEO) databases, we used 253 EGFR-MT LUAD samples and 38 normal lung tissue samples. At the same time, GSE19188 was additionally included to verify the accuracy of the predicted gene. To discover differentially expressed genes (DEGs), the R package “limma” was used. The R packages “WGCNA” and “survival” were used to perform WGCNA and survival analyses, respectively. The functional analysis was carried out with the R package “clusterProfiler.” In total, 1450 EGFR-MT–specific DEGs were found, and 7 tumor-related modules were marked with WGCNA. We found 6 hub genes in DEGs that overlapped with the tumor-related modules, and the overexpression level of B3GNT3 was significantly associated with the worse OS (overall survival) of the EGFR-MT LUAD patients (*p* < 0.05). Functional analysis of the hub genes showed the metabolism and protein synthesis–related terms added value. In conclusion, we used WGCNA to identify hub genes in the development of EGFR-MT LUAD. The established prognostic factors could be used as clinical biomarkers. To confirm the mechanism of those genes in EGFR-MT LUAD, further molecular research is required.

## Introduction

Lung cancer is the most prominent cancer-related cause of death worldwide. Non–small-cell lung carcinoma (NSCLC) accounts for 75–80 percent of all lung cancers and is often detected at an early stage, resulting in a poor prognosis (Liu et al., 2017). Lung adenocarcinoma is the most prevalent form of NSCLC (LUAD) (Yang et al., 2020a).

Significant advances in the understanding of lung cancer, especially LUAD, have been made in recent years. The epidermal growth factor receptor (EGFR) has been identified as an oncoming engine. Especially in Asian lung adenocarcinoma patients, the frequency of EGFR mutations is higher (Devanagari et al., 2015). Treatments for managing EGFR-mutant (EGFR-MT) LUAD included the following: radiation therapy, surgery, chemotherapy, and EGFR tyrosine kinase inhibitors (TKIs) (Hsu et al., 2018). Based on the diagnosis, suitable variations of the three treatment modalities are chosen. Although the overall survival (OS) of EGFR-MT LUAD patients has been significantly improved due to the emergence of TKIs, there are still some critical patients or TKI-resistant patients with limited survival advantages (Yang et al., 2020b).

In the past few decades, high-throughput technologies such as gene chips and gene sequencing have been widely used to identify driver genes and detect important somatic nucleotide polymorphisms, and gene fusions during tumorigenesis, recurrence, and metastasis (Luo et al., 2018; Nahum et al., 2018; O'Farrell et al., 2019). Understanding these genetic alterations may assist in interpreting the molecular mechanism of EGFR-MT LUAD, but the genetic and cytogenetic complexities intrinsic to EGFR-MT LUAD are difficult to uncover because cancer biology is regulated by several factors, including ferroptosis, hypoxia, and tumor microenvironment (Hanahan and Coussens, 2012; Qiu et al., 2017; Gao et al., 2019). It is important to establish a realistic and accurate diagnostic test that can predict the likelihood of EGFR-MT LUAD metastasis or progression.

Structure network algorithms were widely used to identify important nodes in a network by measuring the leadership role of a node based on all of its links (Zeng et al., 2016; Bu et al., 2020). One of the most remarkable methods is weighted correlation network analysis (WGCNA), a scientific tool for explaining the pattern of gene interaction between different samples (Langfelder and Horvath, 2008). It can be used to locate and scan co-expressed gene modules and essential biomarkers. This method has not been used in EGFR-MT LUAD to our knowledge. The aim of our study was to identify novel gene network co-expression modules associated with EGFR-MT LUAD by WGCNA to determine the key signal pathways and genes involved in EGFR-MT LUAD pathogenesis and prognostics.

## Materials and Methods

**FIGURE 1:**
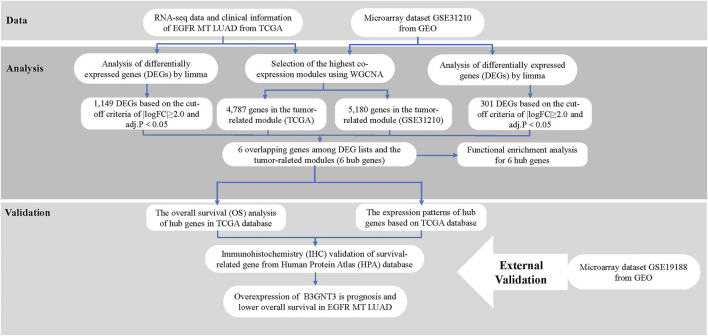
Study design and workflow.

Figure 1 shows the workflow of the analytical key gene extraction pipeline. In the following subsections, we elaborate on each step. In this study, the data of GSE31210 and TCGA were set as the training set for screening key prognostic genes, and the data of GSE19188 were set as the test set to verify the results.

### Data Sources and Data Preprocessing

GSE31210 (Okinawa et al., 2012; Yamauchi et al., 2012) and GSE19188 (Hou et al., 2010) were downloaded from the Gene Expression Omnibus (GEO) database (https://www.ncbi.nlm.nih.gov/geo). The “impute” and “limma” packages were used to supplement the missing data and standardize the two expression profiles. The two data corresponding to clinical information were extracted and integrated for subsequent use.

EGFR-MT LUAD RNA-seq data and corresponding clinical information were downloaded from TCGA (The Cancer Genome Atlas) database (https://portal.gdc.cancer.gov/). 126 EGFR-MT LUAD and 18 normal samples of tissues have been presented. The data were annotated in a human hg38 gene standard track reference transcript. After the count per million (CPM) < 1 gene was filtered using the function space in the “edgeR” package, calculated with gene counts divided according to the gene length, our next analysis was made with 15,213 genes with RPKM values. The detailed information of all the data used in this research is given in Table 1.

**TABLE 1:** Research usage data information.

Data source	Data number	Sample size	Sample type	References	Analytical method
TCGA	None	126	EGFR–mut LUAD	None	DEGs; WGCNA; survival analysis
		18	normal		
GEO	GSE31210	127	EGFR–mut LUAD	13,14	DEGs; WGCNA; survival analysis
		20	Normal		
	GSE19188	45	LUAD	15	Verification of expression level and survival significance
		65	Normal		

### Identification of Tumor-Related Modules With WGCNA

The TCGA-LUAD and GSE31210 gene expression data were built using a “WGCNA” package in the form of genetic co-expression modules in R (Zeng et al., 2016). Soft power *β* = 1 was chosen in both data to create a scale-free network. Next, the adjacency matrix was generated using the following formula: Aid = |Sij|β (aid: adjacency matrix between gene I and gene j, Sij: similarity matrix rendered by Pearson’s association of both gene pairs, β: soft power value) and converted into a topological overlap matrix (TOM) as well as corresponding dissimilarity (1-TOM). A hierarchical clustering dendrogram was formed for the 1-TOM matrix in the subsequent grouping, with a minimum of 50 genes for dendrogram for the same gene expressions, into separate gene co-expression modules. The link between the modules and the details of the clinical characteristics was calculated for tumor-related modules.

### Screen DEGs and Hub Genes Shared With Tumor-Related Modules

We used the *limma* package to screen DEGs of TCGA-EGFR-MT LUAD and GSE31210 (Ritchie et al., 2015). The |log2 (fold change) |>2 and adjust *p* value < 0.05 were set to screen DEGs. The volcano plot of DEGs was visualized by the R package “ggplot2” (Wickham, 2009). Subsequently, genes overlapping in modules linked to tumors harvested as hub gene candidates for later detection were presented as a diagram of Venn using the package “VennDiagram” (Chen and Boutros, 2011).

### Functional Enrichment for Hub Genes

Functional enrichment analysis for hub genes included Gene Ontology (GO) and Kyoto Encyclopedia of Genes and Genomes (KEGG: http://www.kegg.jp/kegg/) pathway enrichment analysis, which were performed for genes by “clusterProfiler” package in R (Yu et al., 2012). An adjusted *p*-value <0.05 was considered significant.

### The Validation of Hub Gene Expression Patterns and Prognostic Values

The expression patterns of the hub gene in various pathological EGFR-MT LA and normal tissue have been tested to validate the reliability of the hub genes. The levels of expression of each hub gene were seen as a box plot graph between EGFR-MT LA and normal tissue. The “survival” package was used to conduct a Kaplan–Meier survival analysis based on the data from TCGA database to explore the correlation between overall survival (OS) and hub genes in patients. For the survival study, only patients who had finished their follow-up period were chosen, and they were split into two classes depending on the median expression value of hub genes. The survival-related hub genes with a log-rank *p* value < 0.05 were regarded as statistically significant. In order to explore the functions and pathways of hub genes which were statistically significant, gene set enrichment analysis (GSEA) was performed in the high-expression and the low-expression groups; gene sets with |NES|>1, NOM *p* < 0.05, were considered to be enrichment significant.

### Microarray Data and the HPA Database Were Used to Verify Protein Expressions of Survival-Related Hub Genes

To verify the expression level and survival significance of the 6 hub genes, we introduced another microarray data of LUAD (GSE19188) for external data verification. Based on the clinical information of GSE19188, the prognostic significance of the 6 hub genes was verified.

At the same time, we used immunohistochemistry (IHC) from the Human Protein Atlas (HPA) database (https://www.proteinatlas.org/) to further verify the protein expression of survival-related genes (Thul and Lindskog, 2018). Also, the protein expression pattern based on IHC is the most commonly used method for detecting the relative position and abundance of proteins in immunotherapy (Maity et al., 2013).

## Results

### Identification of Co-Expression Gene Modules With WGCNA

A total of 7 modules in the TCGA–EGFR-MT LUAD (Supplementary Figure S1A) and 9 modules in the GSE31210 (Supplementary Figure S2A) were identified *via* average linkage clustering (excluding gray modules that were not assigned to any cluster). The results of the module–trait relationships revealed that 3 modules in the TCGA–EGFR-MT LUAD and 4 modules in the GSE6631 were found to have an association with tumor tissues (Supplementary Figures S1B, S2B).

### Screen DEGs and Identification of Hub Genes

**FIGURE 2:**
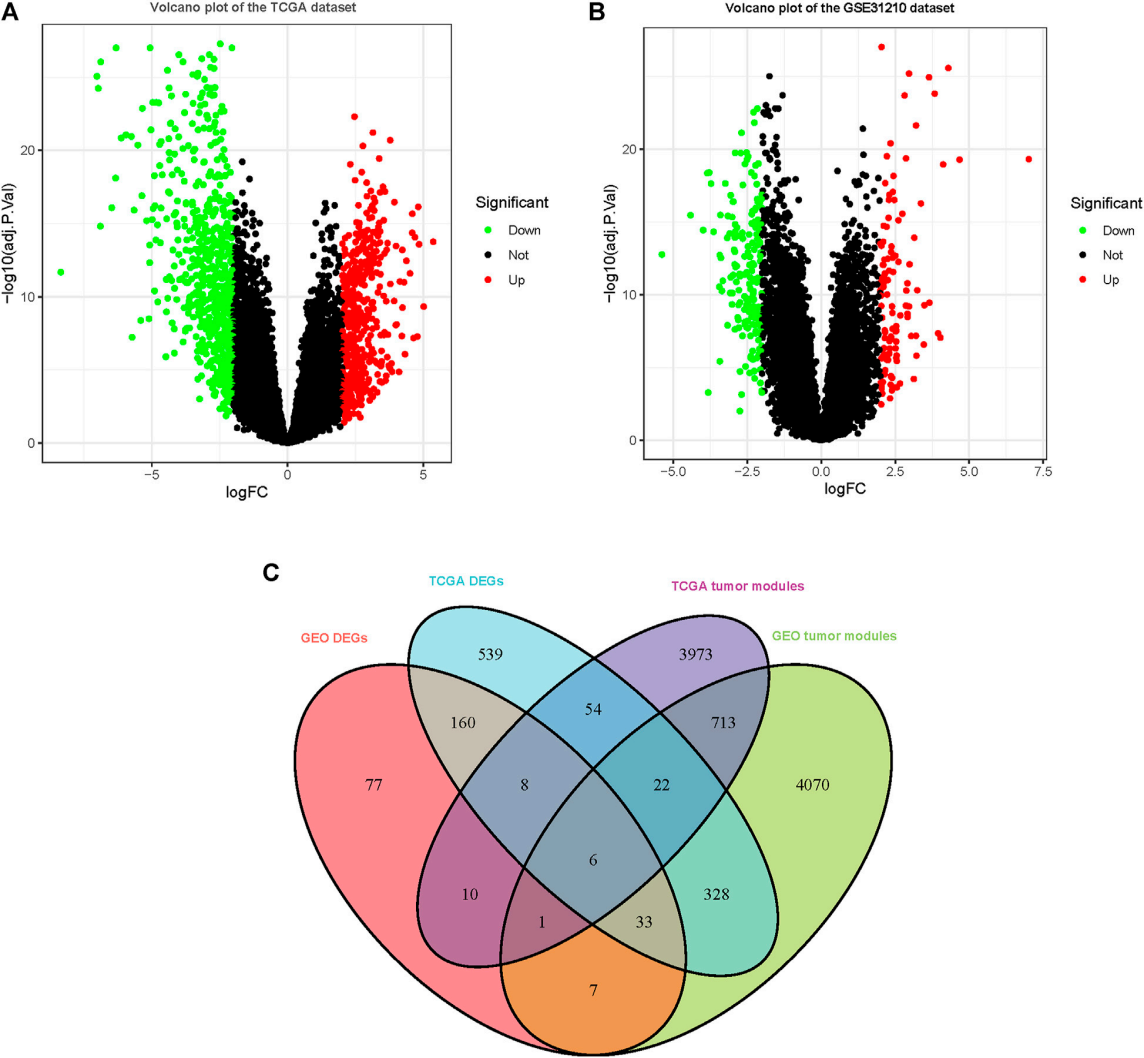
DEGs were observed in TCGA and GSE31210 datasets using |logFC|≥2.0 and adj. *p* < 0.05 as cutoff parameters. **(A)** Volcano plot of DEGs in TCGA dataset. **(B)** Volcano plot of DEGs in the GSE31210 dataset. **(C)** Genes contained in DEGs and tumor-related modules in a Venn diagram. At the intersection of DEGs and modules, there are a total of 6 overlapping genes.

1,149 DEGs in the TCGA dataset (Figure 2A) and 301 DEGs in the GSE31210 dataset (Figure 2B) were defined as deregulated in tumor tissues using a cutoff criterion (log2 (fold change)≥ 2.0 and adj. *p* < 0.05). Subsequently, the extracted 6 genes in DEGs that overlapped with the tumor-related modules including beta-1,3-N-acetylglucosaminyltransferase 3 (B3GNT3), adhering 3 (CDH3), cysteine SN (CST1), zinc finger and BTB domain containing 16 (ZBTB16), keratin 15 (KRT15), and cloth beta (KLB) were selected as the hub genes for subsequent analysis (Figure 2C).

### Functional Enrichment Analysis for Hub Genes

**FIGURE 3:**
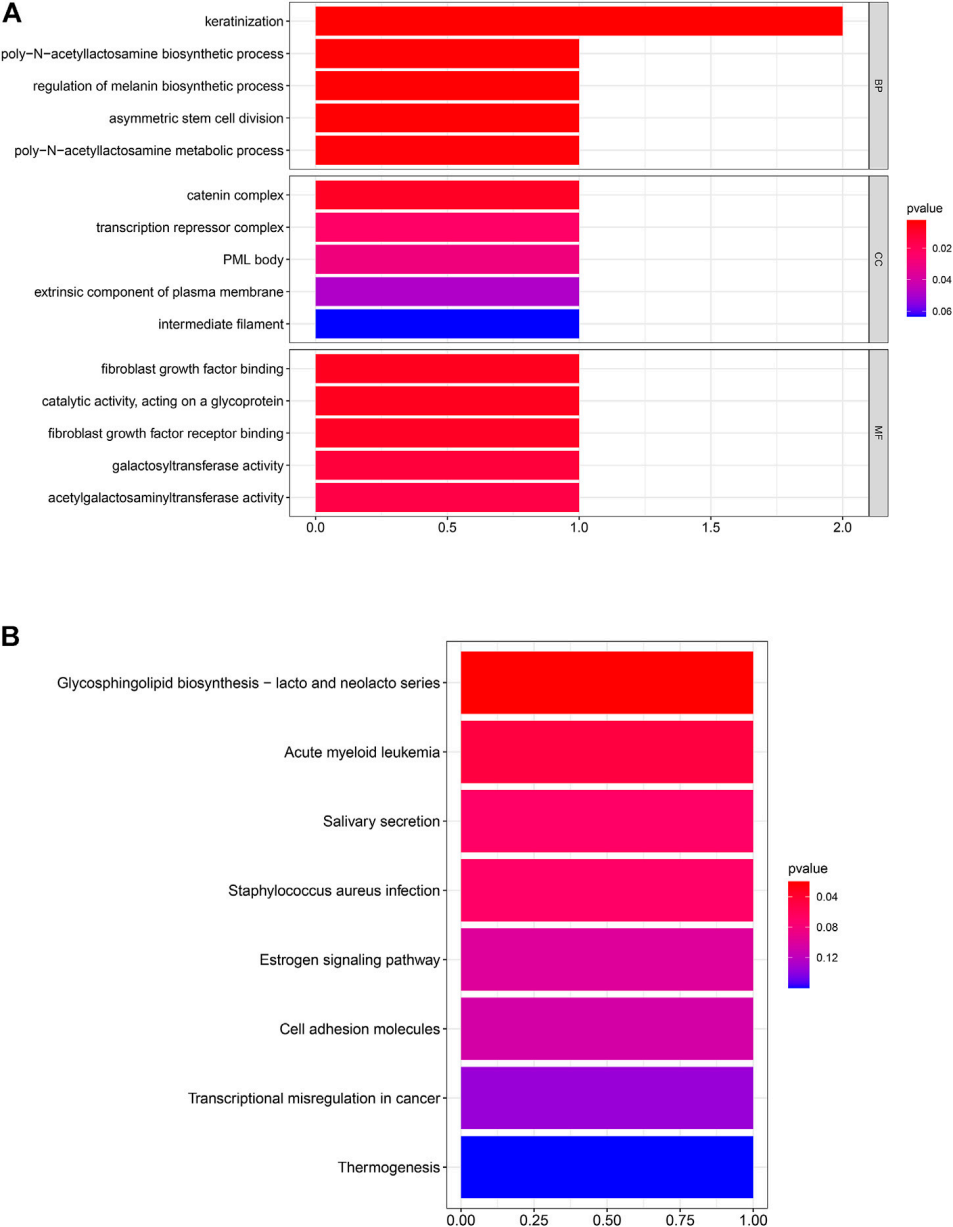
Six hub genes were analyzed for enrichment. The size of the spots represents the gene number, and the color represents the adjusted *p*-values (BH). **(A)** Result of GO enrichment analysis. **(B)** Result of KEGG enrichment analysis.

After the screening of GO enrichment analysis, the top 5 enriched gene sets are shown in Figure 3A. The biological process (BP) of 6 hub genes is mainly enriched in keratinization and the poly-N-acetyllactosamine biosynthetic process. The cellular component (CC) showed that these genes were mainly involved in the catering complex. Moreover, in the molecular function (MF) analysis, fibroblast growth factor binding and fibroblast growth factor receptor binding were suggested to be related to the 6 genes. As shown in Figure 3B, 6 hub genes were enriched in the KEGG pathway of glycosphingolipid biosynthesis—lacto and neglect series.

### Analysis and Verification of the Hub Gene Expression Level and Survival Significance

**FIGURE 4:**
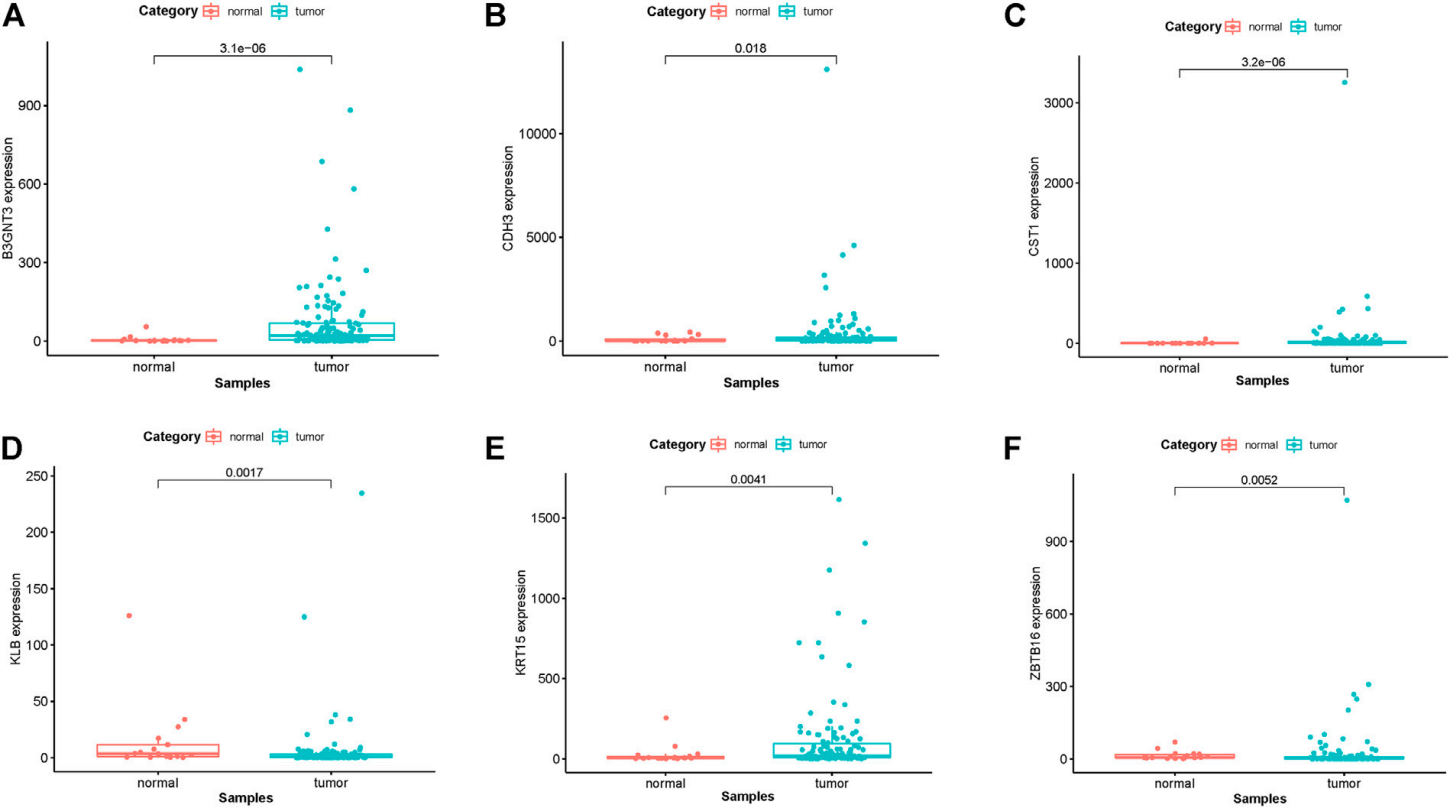
TCGA database was used to verify the expression levels of 6 hub genes in EGFR-MT LUAD and normal tissues. **(A)** Gene expression values of B3GNT3 among samples of TCGA. **(B)** Gene expression values CDH3 among samples of TCGA. **(C)** Gene expression values of CST1 among samples of TCGA. **(D)** Gene expression values of KLB among samples of TCGA. **(E)** Gene expression values of KRT15 among samples of TCGA. **(F)** Gene expression values of ZBTB16 among samples of TCGA.

**FIGURE 5:**
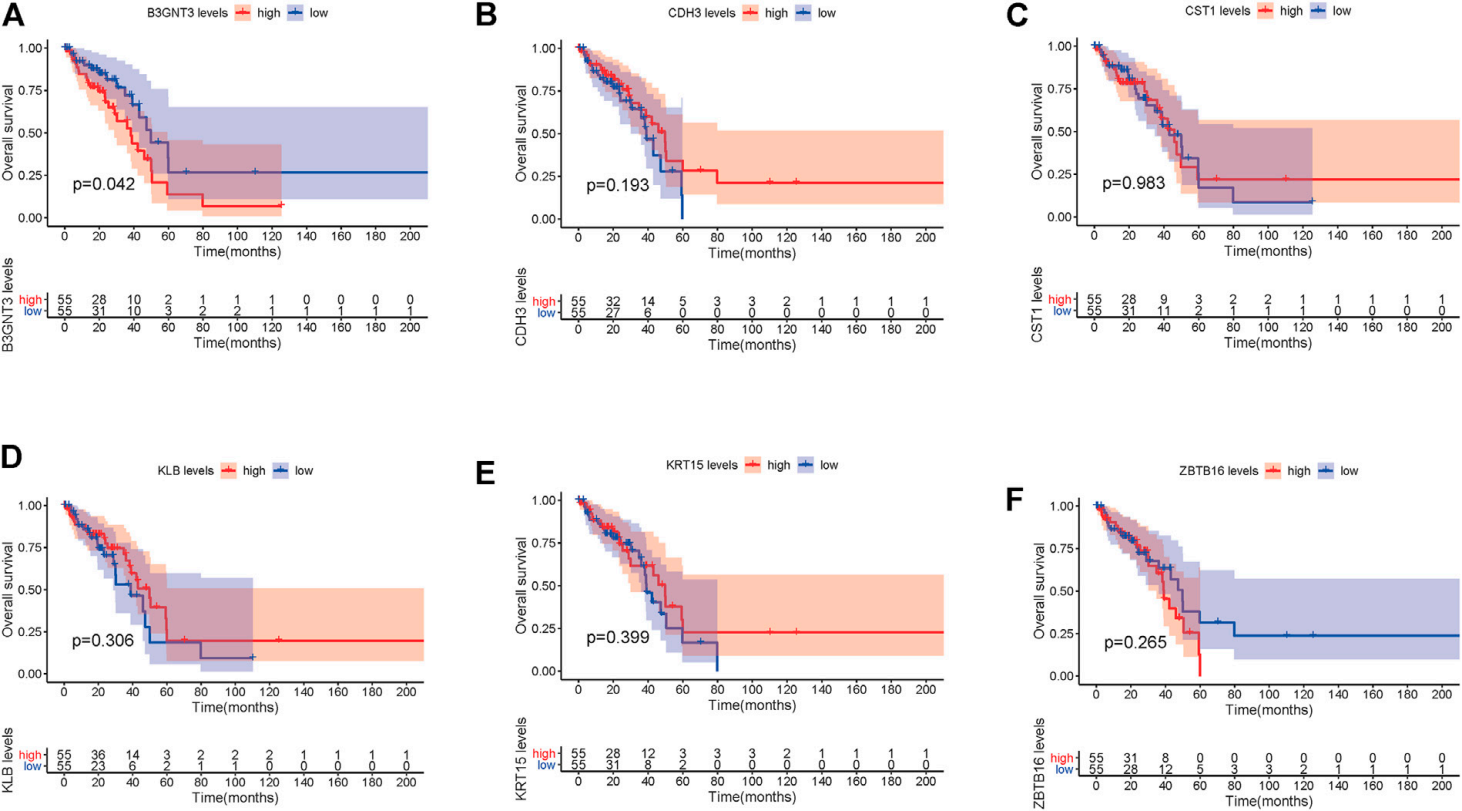
TCGA database was used to look at the overall survival (OS) of 6 hub genes in EGFR-MT LUAD patients. **(A)** Survival analysis for B3GNT3. **(B)** Survival analysis for CDH3. **(C)** Survival analysis for CST1. **(D)** Survival analysis for KLB. **(E)** Survival analysis for KRT15. **(F)** Survival analysis for ZBTB16. The patients were stratified into the high-level group (red) and low-level group (blue) according to the median expression of the gene. Log-rank *p* < 0.05 was considered to be a statistically significant difference.

In EGFR-MT LUAD tissues (RNA-seq data from TCGA), all of the 6 hub genes were found to be substantially downregulated or unchecked, as shown in Figure 4. Furthermore, Kaplan–Meier survival studies of the 6 hub genes showed that B3GNT3 overexpression was substantially correlated with poorer overall survival of EGFR-MT LUAD patients (*p* < 0.05) (Figure 5).

**FIGURE 6:**
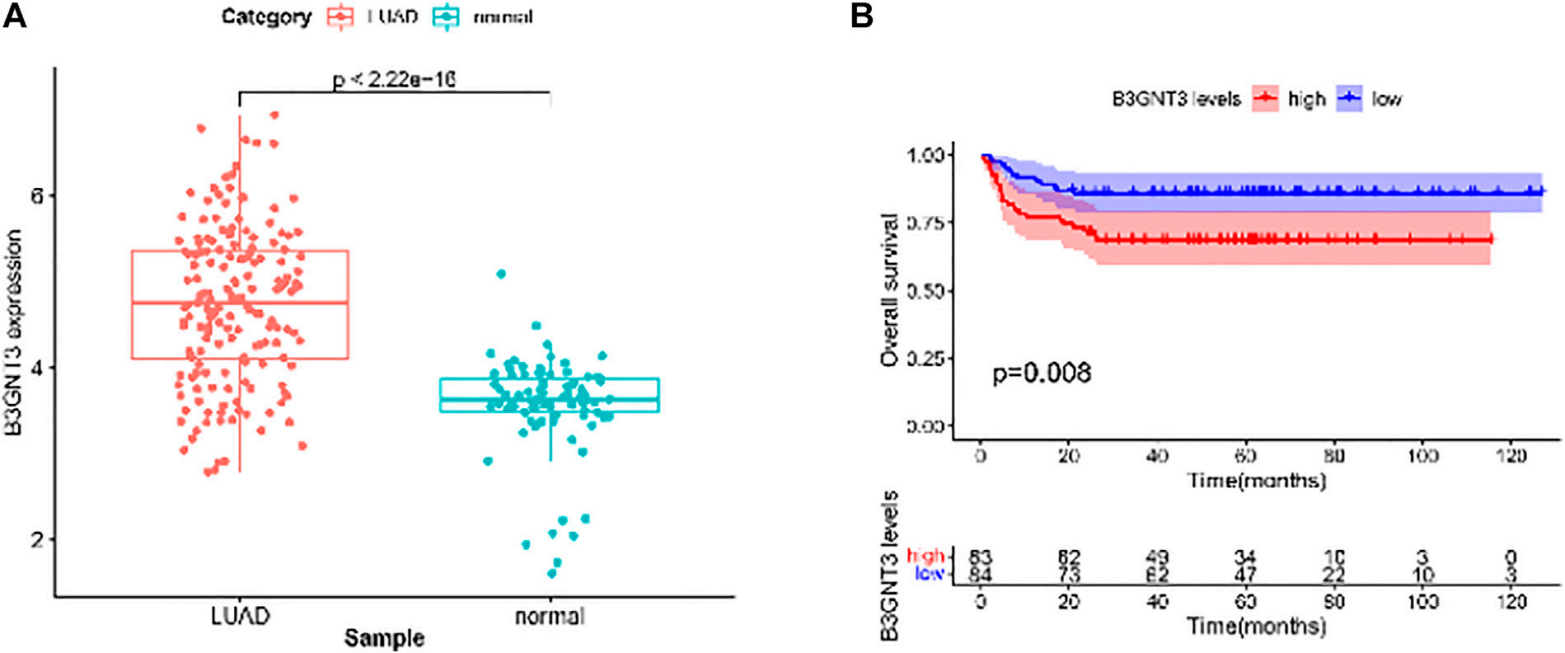
GSE19188 was used to verify the expression levels of 6 hub genes. **(A)** Gene expression values of B3GNT3. The GSE19188 was used to verify the overall survival (OS) of 6 hub genes. **(B)** Survival analysis for B3GNT3. The patients were stratified into the high-level group (red) and low-level group (blue) according to the median expression of the gene. Log-rank *p* < 0.05 was considered to be a statistically significant difference.

**FIGURE 7:**
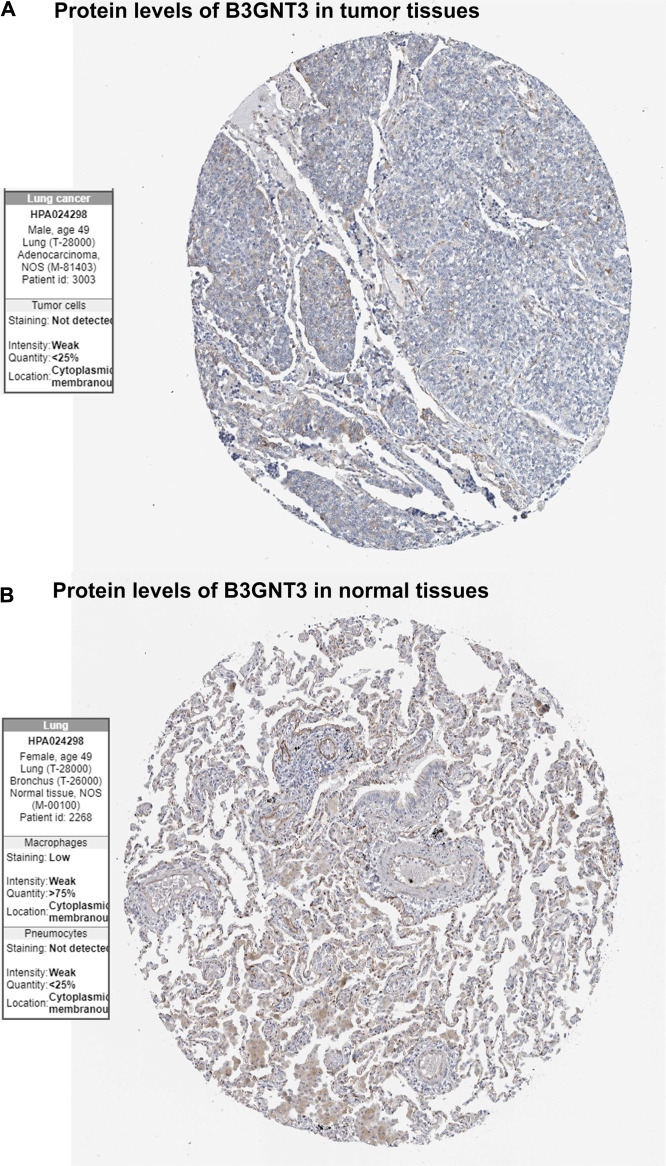
Immunohistochemistry of the B3GNT3 gene in LUAD and normal tissues from the Human Protein Atlas (HPA) database. **(A)** Protein levels of B3GNT3 in LUAD tissues. **(B)** Protein levels of B3GNT3 in normal lung tissues.

GSE19188 was used to verify the expression level and survival significance of the 6 hub genes. It was found that compared with normal lung tissues, the 6 hub genes were significantly inhibited or overexpressed, and the results of B3GNT3 were consistent with the results of RNA-seq data analysis from TCGA (Figure 6). The GSEA enrichment term exhibited that high expression of B3GNT3 was mainly associated with ether lipid metabolism, lysosome, steroid biosynthesis, glycan biosynthesis, and so on (Table 2). According to the HPA database, the protein levels of the B3GNT3 gene were substantially higher in tumor tissues than in normal tissues (Figure 7).

**Tab 2:** GSEA enrichment results for high expression of B3GNT gene.

KEGG pathway name	Size	Enrichment score	Normalized ES	p-val	q-val
Ether lipid metabolism	25	0.578	1.852	0.002	0.479
Lysosome	116	0.605	1.795	0.0191	0.432
Steroid biosynthesis	16	0.711	1.787	0.0113	0.304
Glycan biosynthesis	28	0.563	1.752	0.0116	0.303
Peroxisome	77	0.476	1.721	0.0192	0.313
Amino sugar and nucleotide sugar metabolism	40	0.516	1.623	0.0240	0.552
*Vibrio cholerae* infection	51	0.469	1.612	0.0260	0.506
Pathogenic *Escherichia coli* infection	51	0.487	1.570	0.0456	0.586
Glycerophospholipid metabolism	66	0.385	1.501	0.0260	0.704
Ppar signaling pathway	66	0.399	1.471	0.0403	0.634

Size: The KEGG pathway contains the number of genes in the expression dataset.

## Discussion

The WGCNA is a valuable method for finding highly correlated gene modules. The main module’s intramuscular center could be used for disease detection and prognostication, such as cancer. We use specific DEGs caused by EGFR mutations to perform WGCNA on EGFR-MT LUAD and normal lung samples. We found B3GNT3 correlated with the prognosis of EGFR-MT LUAD patients. Moreover, the functional analysis found these 6 hub genes mainly enriched in keratinization terms and glycosphingolipid biosynthesis—lacto and neglect series pathway.

B3GNT3, also known as acetylglucosaminyltransferase, is a member of the beta-1,3-N-acetylglucosaminyltransferase family (Ho et al., 2013). It plays a dominant role in L-selectin ligand biosynthesis, lymphocyte homing, and lymphocyte trafficking. (Maity et al., 2013). Besides, in early cervical cancer, pancreatic cancer, and neuroblastoma, the level of B3GNT3 mRNA is higher than that of adjacent control tissues (Ho et al., 2013; Zhang et al., 2015; Barkley et al., 2018; Li et al., 2018). B3GNT3 was shown to be upregulated in tumor tissues as opposed to normal tissues in our sample, with a strong link to EGFR-MT LUAD. Higher levels of B3GNT3 have been related to a weak prognosis in patients with NSCLC in previous trials, but it is uncertain which subtype of NSCLC is involved (Gao et al., 2018). That was in line with our survival review results, and our research contributes to the growing body of evidence that B3GNT3 can be used as a diagnostic and prognostic marker for EGFR-MT LUAD.

Although the other 5 hub genes in our study did not suggest significance for the OS of EGFR-MT LUAD patients, studies have confirmed that they are closely related to EGFR-MT LUAD metastasis, recurrence, and drug resistance. Ting et al. found that high CDH3 expression is related to EGFR-TKI resistance (Hsiao et al., 2020a); Cao et al. found that high CST1 expression can be used as a marker for recurrence and metastasis in patients with NSCLC (Cao et al., 2015); Wang et al. found that low expression of ZBTB16 can promote the survival of NSCLC tumor cells and enhance their invasiveness (Wang et al., 2013; Xiao et al., 2015). Our study revealed that these genes are heavily enriched in metabolism-related biological processes such as the poly-N-acetyllactosamine biosynthetic process, glycosphingolipid biosynthesis—lacto and neglect series process. This suggests that they may have an important role in tumor metabolism, to be explored in further studies.

CDH3, a cell adhesion molecule, is associated with the function of cells to bind with other cells and the extracellular matrix (ECM). CDH3 is overexpressed in many malignancies (Kaupmann et al., 1992). In our study, it was also found to be overexpressed in EGFR-MT LUAD. Hsiao et al. (2020b) found that CDH3 overexpression is related to the patients’ EGFR-TKI resistance, and reducing the expression level of CDH3 can increase the sensitivity of EGFR-TKI in patients. Moreover, sCDH3 was positively associated with the tumor stage in non–small-cell lung cancer, although it has not been found to have a significant effect on the prognosis in our study. But these genes’ significance on the metastasis and invasion of EGFR-MT LUAD still needs to be further studied.

CST1 belongs to the type 2 cystatin superfamily, which restricts the proteolytic activities of cysteine proteases. It has been found correlated with multiple tumor metastasis and invasion (Cui et al., 2019). Dai et al. (2017) found that the OS in the low CST1 expression subgroup was significantly superior to the high CST1 expression subgroup. In our study, we found that it is highly expressed in patients with EGFR-MT LUAD, but its effect on the prognosis of patients needs further research to confirm ZBTB16, a member of the Kruppel C2H2-type zinc finger protein family and encodes a zinc finger transcription factor that contains nine Kruppel-type zinc finger domains at the carboxyl terminus. This protein is located in the nucleus, is involved in cell cycle progression, and interacts with a histone deacetylase (Furukawa et al., 2003). Some studies have found that it can be used as a prognostic evaluation marker and potential therapeutic target in reproductive system tumors and Ewing’s sarcoma (Xiao et al., 2016; Xiao et al., 2019), but its role in lung cancer needs further study.

KRT15 is an encoding protein which belongs to the keratin gene family. It has been found to be highly expressed in colon cancer, breast cancer, gastric cancer, and other tumors and has prognostic value (Zhang et al., 2019; Rao et al., 2020; Xu et al., 2020). Ooi et al. (2010) found that this gene is positively expressed in smoking patients with non–small-cell lung cancer and has prognostic value. Its abnormal expression can lead to abnormal airway epithelial damage and repair function, thereby promoting the development of lung cancer.

KLB is a protein-coding gene and mediates binding of fibroblast growth factor (FGF) 21 to the FGF receptor (FGFR). FGF21-KLB-FGFR signaling regulates multiple metabolic systems in the liver (Ji et al., 2019). Andrew et al. (Thompson et al., 2020) found that it is closely related to the increase in the incidence of lung cancer caused by heavy drinking. At the same time, Zhou et al. (2021) found that serum KLB concentration can be used to predict the clinical outcome of NSCLC patients, although in our study, it was found to have an effect on the prognosis of patients. However, more patient omics data are expected to reveal its clinical significance.

As with all research, our work has several limitations. Although we provide a comprehensive bioinformatics analysis to determine the potential diagnostic genes between cancer and normal tissues, it may not be very accurate in evaluating EGFR-MT LUAD patients at every stage. Also, the molecular mechanism of survival-related genes involved in affecting the prognosis of patients with EGFR-MT LUAD needs to be further verified through a series of experiments. In conclusion, our work discovered the important survival-related gene B3GNT3 that can forecast prognosis in EGFR-MT LUAD by combining WGCNA with differential gene expression analysis.

## Data Availability

Publicly available datasets were analyzed in this study. These data can be found here: GSE31210 GSE19188 https://portal.gdc.cancer.gov/.

## Supplementary Material

Supplementary Figure S1 In the TCGA dataset, modules correlated with the clinical trait were identified. **(A)** Co-expression network module cluster dendrogram. Each module was given its color scheme. **(B)** Relationships between modules and traits. Each row represents a color module, and each column represents a clinical characteristic (tumor and normal).

Supplementary Figure S2 Identification of modules in the GSE31210 that are related to the clinical trait. **(A)** Co-expression network module cluster dendrogram. Different colors were applied to each module. **(B)** Relationships between modules and traits. Each row represents a color module, and each column represents a clinical characteristic (tumor and normal).
