# Supplementary figures and images for: Identification of Six Prognostic Genes in EGFR–Mutant Lung Adenocarcinoma Using Structure Network Algorithms
Source: Front Genet. 2021 Nov 16;12:755245. doi: 10.3389/fgene.2021.755245 (PMC8635158; doi:10.3389/fgene.2021.755245)

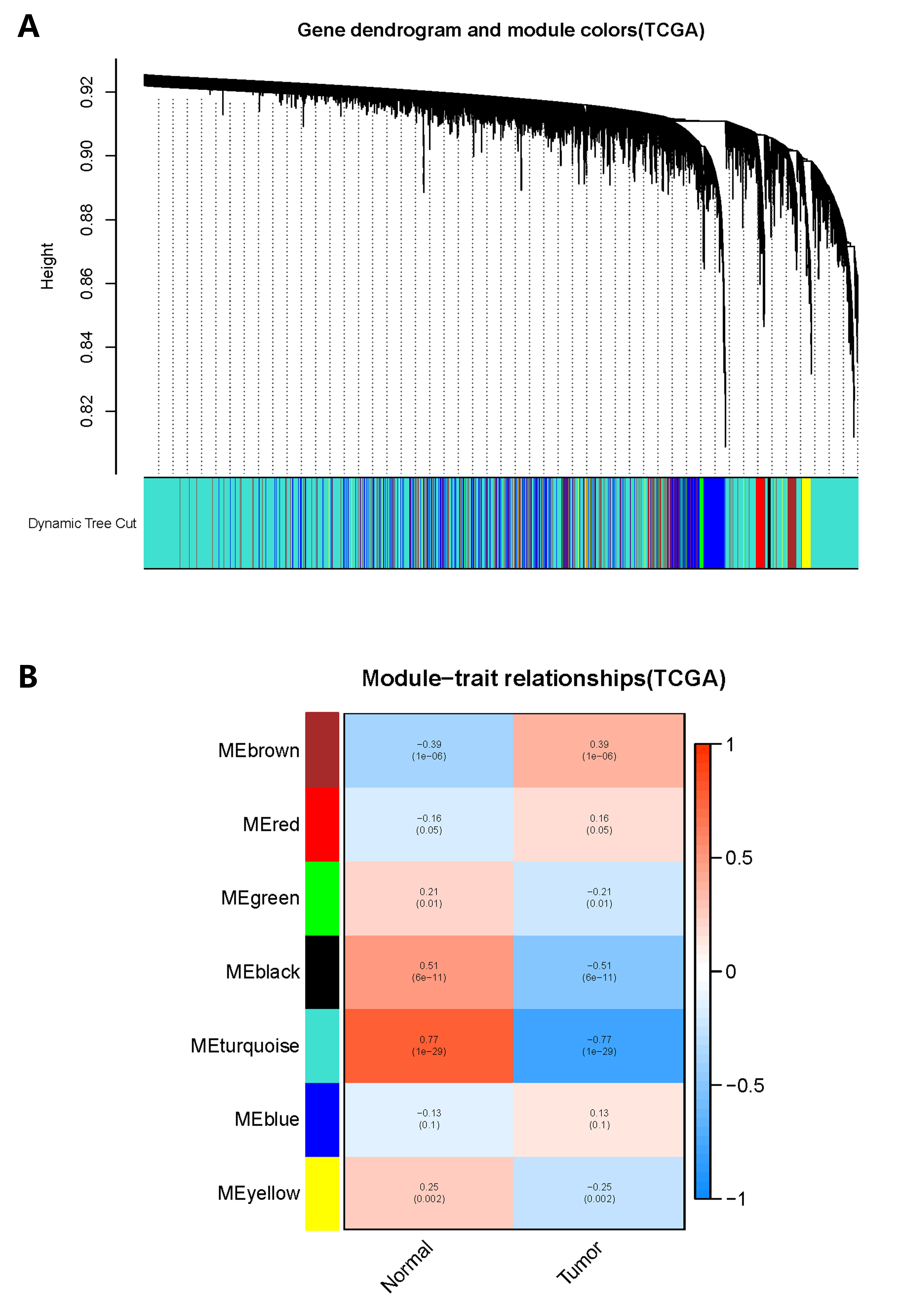

Supplement: Supplementary file 3 [file Image1.JPEG]

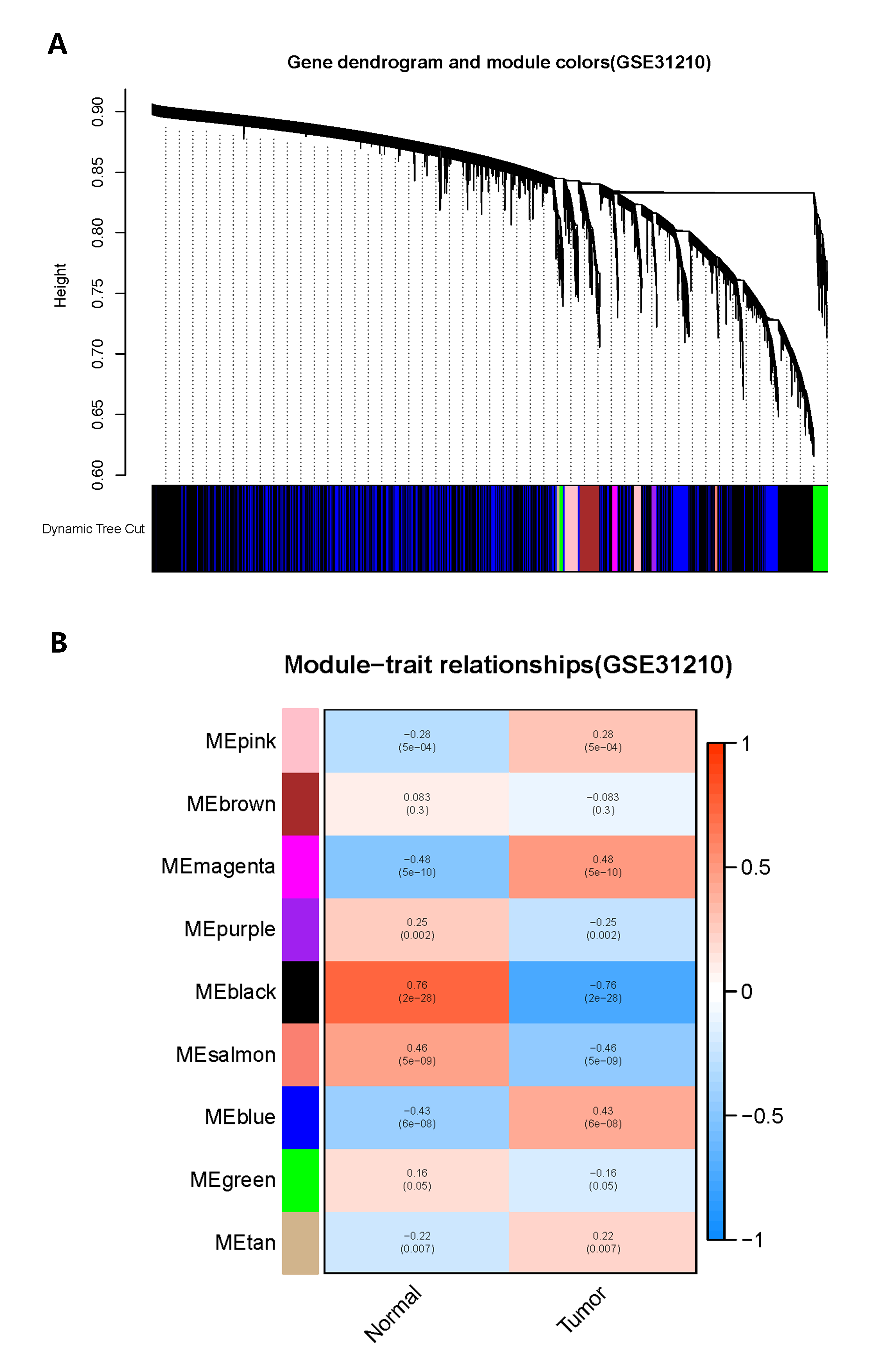

Supplement: Supplementary file 4 [file Image2.JPEG]
